# The role of telepathology in diagnosis of pre-malignant and malignant cervical lesions: Implementation at a tertiary hospital in Northern Tanzania

**DOI:** 10.1371/journal.pone.0266649

**Published:** 2022-04-14

**Authors:** Alex Mremi, Nina Karnøe Bentzer, Bariki Mchome, Joseph Mlay, Jan Blaakær, Vibeke Rasch, Doris Schledermann

**Affiliations:** 1 Department of Pathology, Kilimanjaro Christian Medical Centre, Moshi, Tanzania; 2 Department of Obstetrics and Gynecology, Kilimanjaro Christian Medical University College, Moshi, Tanzania; 3 Department of Pathology, Odense University Hospital, Odense, Denmark; 4 Department of Obstetrics and Gynecology, Kilimanjaro Christian Medical Centre, Moshi, Tanzania; 5 Department of Clinical Research, University of Southern Denmark, Odense, Denmark; 6 Department of Obstetrics and Gynecology, Odense University Hospital, Odense, Denmark; Ruđer Bošković Institute, CROATIA

## Abstract

**Introduction:**

Adequate and timely access to pathology services is a key to scale up cancer control, however, there is an extremely shortage of pathologists in Tanzania. Telepathology (scanned images microscopy) has the potential to increase access to pathology services and it is increasingly being employed for primary diagnosis and consultation services. However, the experience with the use of telepathology in Tanzania is limited. We aimed to investigate the feasibility of using scanned images for primary diagnosis of pre-malignant and malignant cervical lesions by assessing its equivalency to conventional (glass slide) microscopy in Tanzania.

**Methods:**

In this laboratory-based study, assessment of hematoxylin and eosin stained glass slides of 175 cervical biopsies were initially performed conventionally by three pathologists independently. The slides were scanned at x 40 and one to three months later, the scanned images were reviewed by the pathologists in blinded fashion. The agreement between initial and review diagnoses across participating pathologists was described and measured using Cohen’s kappa coefficient (κ).

**Results:**

The overall concordance of diagnoses established on conventional microscopy compared to scanned images across three pathologists was 87.7%; κ = 0.54; CI (0.49–0.57).The overall agreement of diagnoses established by local pathologist on conventional microscopy compared to scanned images was 87.4%; κ = 0.73; CI (0.65–0.79). The concordance of diagnoses established by senior pathologist compared to local pathologist on conventional microscopy and scanned images was 96% and 97.7% respectively. The inter-observer agreement (κ) value were 0.93, CI (0.87–1.00) and 0.94, CI (0.88–1.00) for conventional microscopy and scanned images respectively.

**Conclusions:**

All κ coefficients expressed good intra- and inter-observer agreement, suggesting that telepathology is sufficiently accurate for primary diagnosis in surgical pathology. The discrepancies in interpretation of pre-malignant lesions highlights the importance of p16 immunohistochemistry in definitive diagnosis in these lesions. Sustainability factors including hardware and internet connectivity are essential components to be considered before telepathology may be deemed suitable for widely use in Tanzania.

## Introduction

Cervical cancer is one of the most preventable and treatable malignant diseases. Yet it is the fourth most commonly detected cancer in women worldwide, with more than half a million new cases and 300,000 deaths in 2018 [1]. The disease is disproportionally dispersed, with low- and middle-income countries (LMICs) accounting for more than 90% of the disease burden. This uneven distribution reflects ineffective screening and poor pathological service which are prevailing problems in most LMICs. East Africa is among the regions with highest incidence rate, about ≥42.7 per 100,000 and yet women especially in rural areas do not have access to cervical cancer screening programs [2]. Cervical cancer is the most common cancer (38.4%) and the main cause of female cancer deaths (34.3%) among Tanzanian women [3]. The age-standardized incidence rate is 54 per 100,000 women, which is almost double the average age-standardized rate for Africa (27.6 per 100,000 women) [3, 4]. High incidence and mortality from cervical cancer in Tanzania, suggest an urgent need for improved screening, diagnostic and treatment approaches.

As the number of newly diagnosed cervical cancer patients in Sub-Saharan Africa has increased rapidly, timely access to screening and diagnostic services are necessary pillars for scaling up the cancer control, [5, 6]. However, there is a gap in the complete cancer care cycle in most low-and-middle income countries (LMICs) due to inadequate pathology resources as a result of extreme shortages of pathologists, deficiency of infrastructures and reagents, poor specimen handling and storage [7]. Due to severe shortage of pathologists, many cancer patients do not have timely access to diagnostic service and treatment, and thus die at home or present at the hospital with advanced disease with poor prognosis [7]. Tanzania has limited number of well-trained and experienced pathologists; most of them are general surgical pathologists who are not sub-specialized, working in teaching hospitals in large cities [8]. Likewise, there is a challenge in specimens transport, costs and long turn-around time which altogether contribute to the delayed diagnosis [7, 8]. Pathologists in LMICs may have difficulties in diagnosing rare, complex or challenging cancer cases because in these settings advanced diagnostic tests such as molecular or immunohistochemistry are usually not readily available. Thus, error in pathology diagnosis is not uncommon, which often may result in wrong treatment [9].

Telepathology is the practice of pathology using telecommunications to transmit digital images and data between two or more sites remotely located from each other [10]. The use of telepathology increases access to specialized pathology services, and the technology can be used in routine surgical pathology, consultations, quality assurance, education and research [10–12]. However, there is scanty data on the use of telepathology to support cancer care in Tanzania [13–15]. We therefore conceived the present study to investigate the feasibility and the role of implementing telepathology by assessing the agreement in the diagnoses of cervical biopsies made conventionally on glass slides and on scanned images telepathology platform across the participating pathologists.

## Material and methods

### Study design and study setting

This was a cross-sectional study conducted from January to December 2020 at Kilimanjaro Christian Medical Center (KCMC), which is one of the major four referral and consultant hospitals in Tanzania.

At the end of 2019, Tanzania’s population was estimated to be at 55.9 million, the KCMC hospital serves about 17 million people in Northern part of the country and Kilimanjaro region [16]. The hospital is a University Teaching Hospital for Kilimanjaro Christian Medical University College (KCMUCo) and it offers both general and specialized care. For many years, the Pathology Department of KCMC, had no local pathologists; pathology services were supported by occasional visiting pathologists from Radboud University Medical Center, Netherlands. In the year 2016, two junior local (Tanzanians) pathologists joined the Pathology Department. The first author (AM) is one of the two local pathologists.

### Telepathology platform

**Fig 1:**
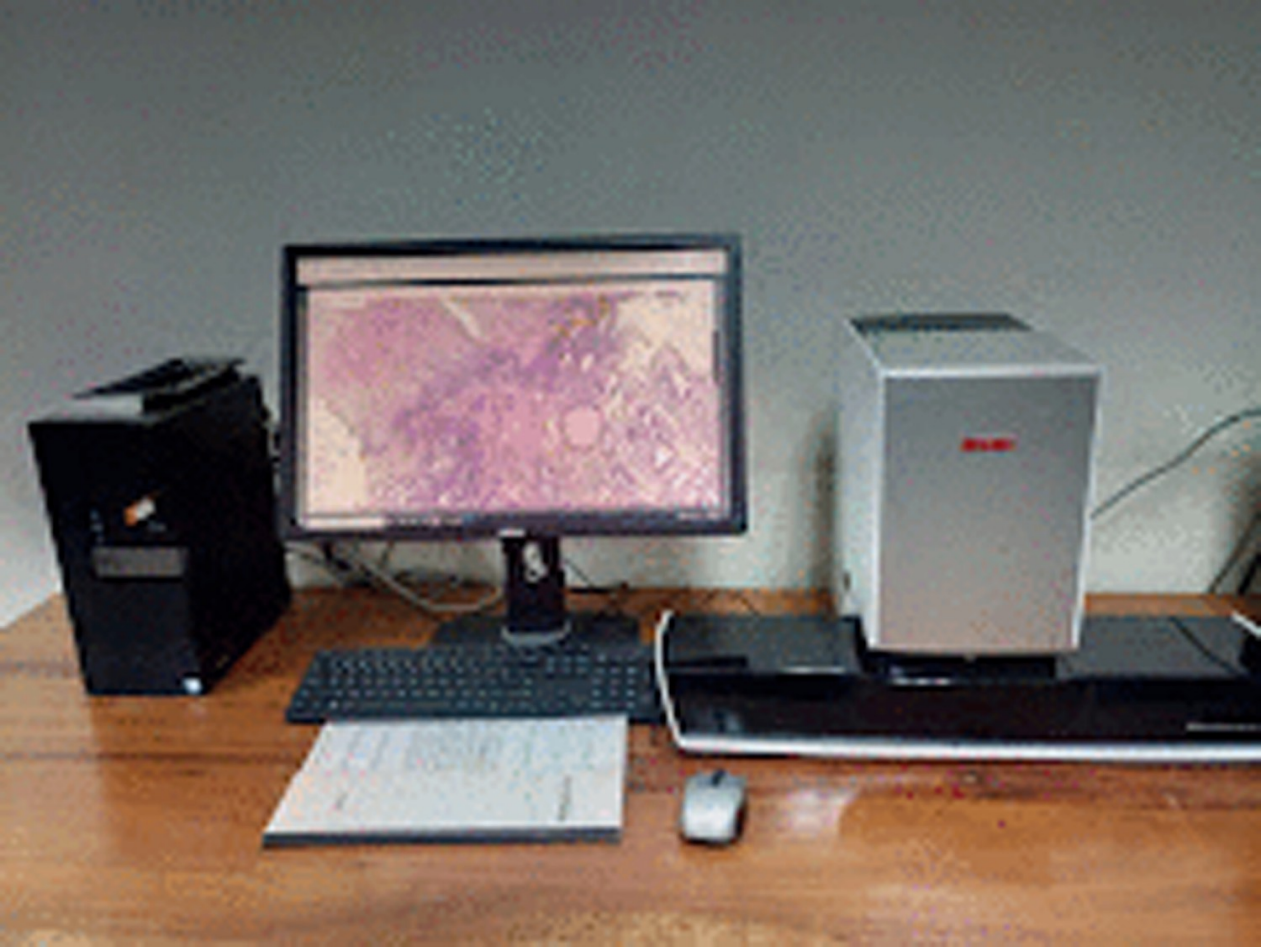
A photograph of the Motic telepathology platform.

**Fig 2:**
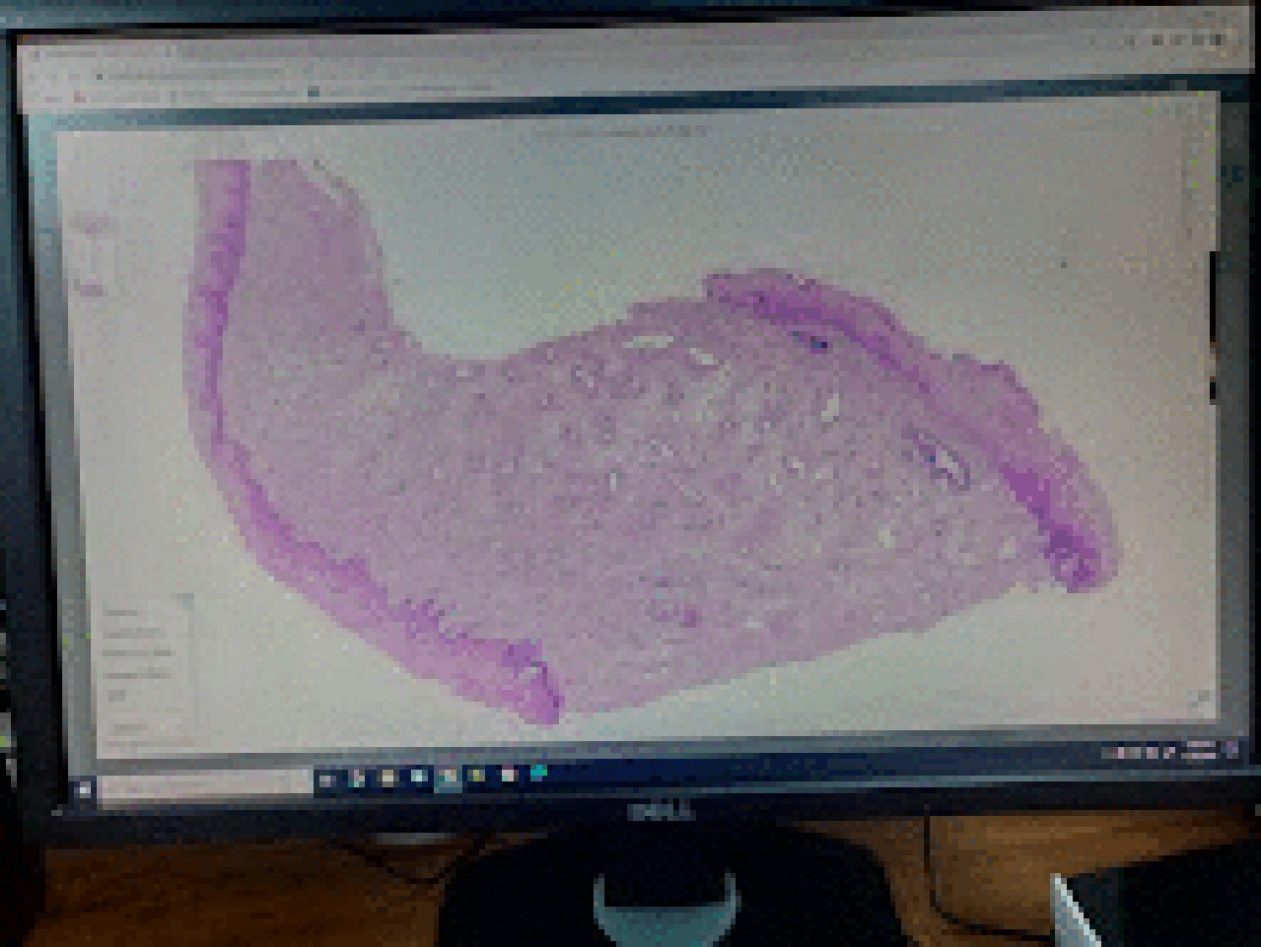
Telepathology working station portal illustrating the digital image of a normal cervical biopsy, H&E staining 20 x original magnification.

In response to increasing demands of timely and reliable histological diagnosis of malignant diseases, KCMC acquired a whole slide image scanning (WSI) equipment which was installed in November 2019. The system (Motic EasyScan, USA), is a website based tele-consultation platform (www.med3.motic.com), developed for capacity building on cancer diagnosis and management in resources limited setting (Figs 1 and 2). After successful installation and training of the KCMC Pathology Department staff, consultant and sub-specialized pathologists were registered and linked in the platform. Continuous communication between KCMC pathologists, Information Technology (IT) personnel and consultant telepathologists was made electronically to address any raised issues with regard to the quality of the images and troubleshooting challenges that hindered the smooth system workflow of the platform.

### Recruitment procedure

A detailed participants enrollment procedure has been described elsewhere [5]. Briefly, women residing in rural Kilimanjaro were invited via public announcements to attend a community based cervical cancer screening with HPVself-sampling using the Evalyn Brush^®^. Authorization to conduct the study was obtained from the local government management committees as well as the community stakeholders. Two experienced reproductive health nurses informed women who showed up for screening about the study objectives and eligibility criteria, and they received written informed consent from all participants. *Care*HPV was used to test the presence of high risk HPV infection. Participants found with positive HPV test were recalled for further investigations which included cervical visual inspection with acetic acid (VIA), Pap smear and cervical biopsies. Consecutive biopsies obtained from the participants were routinely processed and stained with Hematoxylin and Eosin (H&E) at the Pathology Department of KCMC.

### Data collection

H&E stained glass slides of recruited cases were first interpreted conventionally by light microscope, (Olympus BX43 F, Japan). After primary reading the glass slides, they were scanned at 40X original magnification using the Motic whole slide scanner. The images were stored in a local server. Each scanned case was accompanied with relevant anonymized clinical information of the participant. After a washout period of at least three months of the primary glass slide reading (conventional microscopy), the local pathologist reviewed the scanned slides on a 24-inch monitor (Dell, Round Rock, Texas).

The microscopic glass slides of the study cases were then shipped to Odense University Hospital (OUH), Denmark where two pathologists (an experienced senior gynecologic pathologist and a junior surgical pathologist) reviewed them independently. After a wash-out period of at least one month of reviewing the glass slides, the OUH pathologists reviewed the scanned images on their personal computer screens in a blinded fashion.

### Data analysis

Results for categorical variables were expressed as absolute numbers and percentages and 95% Confidence Intervals (CI). The agreement between arbitrary pairs of observers (inter-observer agreement) was measured by kappa statistics (κ). Kappa is an index of agreement over and above that which is expected by chance alone and is scored as a number between 0 and 1 [17]. Here, intra-observer reliability describes whether pathological diagnoses rendered using telepathology platform (scanned images) are comparable (non-inferior) to diagnoses made by conventional microscopy e.g. degree of agreement between the two diagnostic methods. Inter-observer agreement describes the degree of agreement across pathologists. Inter-observer reliability demonstrates the level of agreement in establishing diagnoses on conventional microscopy and scanned images across the pathologists. A nomenclature recommended by Landis-Koch was adopted for interpreting the strength of agreement (κ); values >0.75 are regarded as excellent agreement beyond chance, values between 0.40 and 0.75 as fair to good agreement beyond chance and values <0.40 as poor agreement beyond chance [18]. To test the quality of response rates from glass slides and scanned images diagnoses, McNemar’s test was used. An effect was considered statistically significant if the p-value of its corresponding test statistic was 5% (p <0.05).

Differences in diagnoses established across the pathologists were evaluated and scored on a three-point scale: “Discordance” corresponded to cases with a difference in diagnosis that would be associated with a difference in patient care, e.g. when initial diagnosis was normal cervix while review diagnosis was cancer; normal versus CIN2, normal versus CIN3. “Partial concordance” corresponded to cases with a minor discrepancy in diagnosis that may not be associated with a difference in patient care, e.g. normal versus CIN1or CIN2 versus CIN3. “Concordance” corresponded to cases where both observers gave identical diagnoses, (S1 Appendix). The statistical analyses were performed using the software packages StatXact version 16 (MathSoft, Inc, Seattle, WA, USA).

### Final gold standard diagnosis and ethical considerations

In case of discrepancy, the diagnosis established on conventional microscopy by the senior pathologist was considered as gold standard. The local pathologist was legally responsible for the final diagnoses and thus, signed out a final report for each case. Diagnoses rendered by pathologists from OUH were regarded as second opinions since OUH pathologists are not licensed to practice medicine in Tanzania. The three pathologists did not discuss the diagnostic criteria prior to the study but referred to the WHO Classification [19].

The study was approved by the College Research Ethics Review Committee at Kilimanjaro Christian Medical University College and the National Institute for Medical Research of Tanzania; with approval certificates numbers of 2401 and NIMR/HQ/R.8a/Vol.IX/3093 respectively.

## Results

A total of 175 H&E stained glass slides and their respective 175 scanned images were included in this analysis. The diagnoses were grouped into five categories: normal, CIN1, CIN2, CIN3 and cervical cancer. When diagnoses established by the local pathologist on conventional light microscopy were compared to scanned images, complete agreement was observed in 87.4% of the cases with intraobserver kappa statistic strength (ƙ) of 0.73; CI (0.65–0.79). However, the agreement varied widely among diagnostic categories. Complete agreement was 94.5%, 60%, 25%, 60% and 50% for the normal, CIN1, CIN2, CIN3 and cancer diagnostic categories respectively. For the senior gynecologic pathologist, the overall complete agreement between conventional light microscopy and telepathology was 85.7%; with intraobserver variability (ƙ) value of 0.76; CI (0.69–0.82). Agreement among diagnostic categories were 93.9%, 70.5%, 42.8%, 64.3% and 57.1% for the normal, CIN1, CIN2, CIN3 and cancer diagnostic categories respectively. On the other hand, the overall agreement of diagnoses established by the junior pathologist on conventional microscopy compared to scanned images was 90.9%. The intraobserver variability (ƙ) value for the junior pathologist was 0.73; CI (0.65–0.79). Agreement among diagnostic categories were 97.1%, 78.6%, 25%, 75% and 50% for the normal, CIN1, CIN2, CIN3 and cancer diagnostic categories respectively, Table 1.

**Table 1:** Agreement of diagnoses established on telepathology compared to conventional microscopy across the pathologists.

Local pathologist	Telepathology						
Conventional microscopy	Normal	CIN1	CIN2	CIN3	Cancer	Total	Concordance
Normal	**121(94.5%)**	4(3.1%)	1(0.8%)	2(1.6%)	0 (0%)	128	121 (94.5%)
CIN1	5(25%)	**12(60%)**	2(10%)	1(5%)	0(0%)	20	12 (60%
CIN2	2(50%)	0(0%)	**1(25%)**	1(25%)	0(0%)	4	1 (25%)
CIN3	4(26.7%)	1(6.7%)	0(0%)	**9(60%)**	1(6.6%)	15	11 (60%)
Cancer	1(12.5%)	0(0%)	0(0%)	3(37.5%)	**4(50%)**	8	4 (50%)
Total	133(76%)	17(9.7%)	4(2.3%)	16(9.1%)	5(2.9%)	175	153(87.4%)
Senior pathologist	Telepathology						
Conventional microscopy	Normal	CIN1	CIN2	CIN3	Cancer	Total	Concordance
Normal	**122(93.9%)**	2(1.5%)	2(1.5%)	4(3.1%)	0(0%)	130	122(93.9%)
CIN1	3(17.7%)	**12(70.5%)**	1(5.9%)	1(5.9%)	0(05)	17	12(70.5%)
CIN2	2(28.6%)	2(28.6%)	**3(42.8%)**	0(0%)	0(0%)	7	3(42.8%)
CIN3	2(14.3%)	0(0%)	3(21.4%)	**9(64.3%)**	0(0%)	14	9(64.3%)
Cancer	1(14.3%)	0(0%)	0(0%)	2(28.5%)	**4(57.1%)**	7	4(57.1%)
Total	130(74.3%)	16(9.1%)	9(5.1%)	16(9.1%)	4(2.3%)	175	150(85.7%)
Junior pathologist	Telepathology						
Conventional microscopy	Normal	CIN1	CIN2	CIN3	Cancer	Total	Concordance
Normal	**135(97.1%)**	2(1.4%)	2(1.4%)	0(0%)	0(0%)	139	**135(97.1%)**
CIN1	1(7.1%)	**11(78.6%)**	0(0%)	2(14.3%)	0(0%)	14	**11(78.6%)**
CIN2	1(25%)	0(0%)	**1(25%)**	2(50%)	0(0%)	4	**1(25%)**
CIN3	1(8.3%)	1(8.3%)	1(8.3%)	**9(75%)**	0(0%)	12	**9(75%)**
Cancer	2(33.3%)	0(0%)	0(0%)	1(16.7%)	**3(50%)**	6	**3(50%)**
Total	140(80%)	14(8%)	4(2.3%)	14(8%)	3(1.7%)	175	**159(90.9%)**

When diagnoses established on conventional microscopy by the senior pathologist were compared to the local pathologist, the overall agreement was 96%; with interobserver value (k) of 0.93; CI (0.87–1.00). Across diagnostic categories, the rate of agreement were 97.7%, 100%, 57.1%, 92.3% and 87.5% for the normal, CIN1, CIN2, CIN3 and cancer respectively. Similarly, on telepathology platform, the overall concordance for diagnoses established by senior pathologist compared to local pathologist was 96.6%. The interobserver reliability (k) value was 0.94; CI (0.88–1.00). Similarly, the concordance rates were 98.4%, 94.7%, 71.4%, 93.3% and 100% for the normal, CIN1, CIN2, CIN3 and cancer respectively (Table 2).

**Table 2:** Agreement between senior and local pathologists for diagnoses established on conventional microscopy and telepathology.

Concentional microscopy	Local pathologist						
Senior pathologist	Normal	CIN1	CIN2	CIN3	Cancer	Total	Concordance
Normal	**127(97.7%)**	2(1.5%)	0(0%)	1(0.8%)	0(0%)	130	127 (97.7%)
CIN1	0(0%)	**17(100%)**	0(0%)	0(0%)	0(0%)	17	17 (100%)
CIN2	0(0%)	1(14.3%)	**4(57.1%)**	1(14.3%)	1(14.3%)	7	4 (57.1%)
CIN3	1(7.7%)	0(0%)	0(0%)	**12(92.3%)**	0(0%)	13	12 (92.3%)
Cancer	0(0%)	0(0%)	0(0%)	1(12.5%)	**7(87.5%)**	8	7 (87.5%)
Total	128(73.1%)	20(11.4%)	4(2.3%)	15(8.6%)	8(4.6%)	175	168 (96%)
Telepathology	Local pathologist						
Senior pathologist	Normal	CIN1	CIN2	CIN3	Cancer	Total	Concordance
Normal	**128(98.5%)**	2(1.5%)	0(%)	0(%)	0(%)	130	128 (98.4)
CIN1	1(5.3%)	**18(94.7%)**	0(0%)	0(0%)	0(0%)	19	18 (94.7%)
CIN2	0(0%)	1(14.3%)	**4(57.1%)**	2(0%)	0(0%)	7	5 (71.4%)
CIN3	0(0%)	0(0%)	0(0%)	**14(93.3%)**	1(6.7%)	15	14 (93.3%)
Cancer	0(0%)	0(0%)	0(0%)	0(0%)	**4(100%)**	4	4 (100%)
Total	129(73.7%)	21(12%)	4(2.3%)	16(9.1%)	5(2.9%)	175	169 (96.6%)

To assess the agreement of diagnoses established on glass slides compared to the scanned images across the three pathologists, a symmetric agreement matrix was created in which each diagnosis from each pathologist was compared with the corresponding diagnoses made by the other two observers. This matrix resulted in 1050 paired comparisons in total (3 observers, 2 comparisons, and 175 slides. The overall concordance across the three pathologists was 87.7%; k-value 0.54; CI (0.49–0.57). Across the diagnostic category, the concordance rates were 93.7%, 62.7%, 38.5%, 71.1% and 90.6% for normal, CIN1, CIN2, CIN3 and cancer respectively, the data summarized in Table 3.

**Table 3:** Agreement of diagnoses established on conventional microscopy compared to telepathology across three pathologists.

Telepathology	Conventional microscopy						
	Normal	CIN1	CIN2	CIN3	Cancer	Total	Concordance
Normal	**749(93.7%)**	36(4.5%)	5(0.6%)	8(1.0%)	1(0.1%)	799	749 (93.7%)
CIN1	32(29.1%)	**69(62.7%)**	2(1.8%)	6(5.5%)	1(0.9%)	110	69 (62.7%)
CIN2	4(15.4%)	4(15.4%)	**10(38.5%)**	8(30.8%)	0(0%)	26	10 (38.5%)
CIN3	8(9.6%)	3(3.6%)	3(3.6%)	**64(77.1%)**	5(6.1%)	83	64 (71.1%)
Cancer	0(0%)	0(0%)	0(0%)	3(9.4%)	**29(90.6%)**	32	29 (90.6%)
Total	793(75.5%)	112(10.7%)	20(1.9%)	89(8.5%)	36(3.4%)	1050	921 (87.7%)

Overall Cohen’s kappa coefficient (ƙ) = 0.54; 95% Confidence Interval = (0.49–0.57).

When diagnoses established on conventional microscopy were classified on a three-point scale; the rate of concordance was 66.3%, 66.9% and 82.3% between the three pathologists, and the rate of discordance was 2.3%, 4.6% and 1.7%. On scanned images, the rate of concordance was nearly comparable among the three pathologists with 73.3% for senior versus local pathologist, 76.6% for senior versus junior pathologist and 78.3% for junior versus local pathologist, and the rate of discordance was 1.1%, 3.4% and 4.0% (Table 4).

**Table 4:** Three-point scale agreement of diagnoses established by senior pathologist compared to local and junior pathologists on conventional and scanned images.

Conventional microscopy	Senior versus local pathologist		Senior versus junior pathologist		Junior versus local pathologist	
	Number	Percentages	Number	Percentages	Number	Percentages
Concordance	116	66.3%	117	66.9%	144	82.3%
Partial concordance	55	31.4%	50	28.6%	28	16.0%
Discordance	4	2.3%	8	4.6%	3	1.7%
Total	175	100%	175	100%	175	100%
Telepathology	Senior versus local pathologist		Senior verus junior pathologist		Junior versus local pathologist	
	Number	Percentages	Number	Percentages	Number	Percentages
Concordance	129	73.7%	134	76.6%	137	78.3%
Partial concordance	44	25.1%	35	20%	31	17.7%
Discordance	2	1.1%	6	3.4%	7	4%
Total	175	100%	175	100%	175	100%

## Discussion

In this study, good agreement (87.4%; κ = 0.73) was found when comparing diagnoses established on conventional microscopy and scanned images by local pathologist; and across all 3 study pathologists, the overall concordance between conventional microscopy and scanned images was high (87.7%).

The high intraobserver agreement of histological evaluation of cervical biopsies by the participating pathologists between conventional and scanned images shown in this study suggests that the diagnoses of cervical premalignant and malignant lesions are highly reproducible using both glass and digital formats. This implies that the use of digital images can be used for primary diagnosis and thus address the challenge of shortage of pathologists in Tanzania by providing alternative means for the health facilities without pathologists to access pathology healthcare services. Similar findings have been reported in a study conducted in Spain which demonstrated almost perfect agreement between the diagnoses established on conventional microscopy and scanned images, (κ = 0.91; CI (0.88–0.95)) [20].

The results of our study are comparable with studies on feasibility and validation of telepathology conducted in Africa which have reported a slightly higher concordance rate between diagnoses established on conventional microscopy and scanned images. These studies reported good feasibility regarding the use of telepathology and also found scanned images to be of good quality, however internet speed was found to be a limiting factor. Unlike the current study which focused on cervical specimens, the former studies involved a wide spectrum of specimens (different organ systems); and immunohistochemistry tests were used whenever it was necessary [21, 22]. The findings of our study suggest that through telepathology, specific or definitive diagnoses can be achieved; and the discrepancies observed in our study are within the range of generally acceptable interobserver variability in surgical pathology practice [23].

In this study, the overall concordance for the diagnoses established on conventional microscopy compared to scanned images across the three pathologists was excellent (87.7%). All participating pathologists established nearly comparable intra-observer diagnostic accuracy between conventional and scanned images suggesting that telepathology can potentially be a reliable tool to support primary cancer diagnosis in Tanzania. However, across diagnostic categories, relatively low concordances were observed in CIN2 lesions (38.5%), but it is well known that this diagnosis in particular has low reproducibility. Moreover, our study was not primarily intended to compare diagnostic capabilities between pathologists in Tanzania and Denmark, but rather to demonstrate the feasibility and role of implementing telepathology in supporting healthcare in Tanzania. Factors which may explain the observed discrepancy include poor quality of the glass slides or scanned images in some cases, and borderline lesions which can be challenging to interpret without the use of immunohistochemistry. High quality slides and images preparation are necessary for optimizing diagnostic accuracy for both conventional and digitalized slides. This is among the key areas that need to be considered in digital pathology.

When the diagnoses established on convention microscopy by local pathologist are compared to the senior pathologist, four discrepant cases were identified and diagnosed as CIN3/normal, cancer/CIN3, CIN2/cancer and CIN2/CIN3 whereas only two discrepant cases were found on the scanned slides diagnosed as CIN2/CIN3 and CIN3/cancer. The majority of the partial discordances (72.7%) were related to discrepancies in the diagnosis of CIN1 lesions versus normal/reactive cervical epithelium which has minor clinical implications. Across diagnostic categories, these partial discrepancies that are without consequences for the clinical care, resulted in a lower κ value than the overall value. Studies using conventional microscopy have reported a substantial variation among and within pathologists’ observers in the interpretation of various histopathological lesions on H&E-stained tissue sections [24]. The use of p16 immunohistochemistry has been associated with reduction of interobserver disagreement in the interpretation of cervical lesions and improves detection of high grade precancerous lesions associated with HPV infection; as p16 is overexpressed in almost all high grade lesions and negative or normal in reactive lesions. Recently, p16 has been recommended in the evaluation of cervical biopsies since it reduces the interobserver variability, particularly in cases of professional disagreement [25, 26].

In this study, both the local and OUH pathologists had little or no previous experience in the use of telepathology, however this did not affect the reproducibility. This may suggest that if telepathology is widely introduced in Tanzania, only minor difficulties should be expected. Tanzania has a shortage of more than 534 medical specialists, a situation which is worse in rural areas [27]. Yet, the current rate of training suggests that it may take centuries before adequate number of specialist pathologists is obtained [28]. In addition, as a developing country, challenges in medical services are likely to exist for a long time to come. At present, it is practically impossible for many patients to obtain pathology services in a timely manner since the services are only available in tertiary hospitals [8]. Thus, in order to scale up cancer control, implementation of telepathology should be considered. Telepathology infrastructures may facilitate timely access to diagnostic and specialized healthcare services through networking. Moreover, telepathology can reduce travel costs for a number of patients who are referred to high level referral hospitals for diagnostic services [29–31]. However, with the increasing cancer burden, telepathology cannot be an ultimate solution for the shortage of pathologists. The government and other stakeholders in healthcare should also invest in training new pathologists who are able to provide essential and specialized services in the country.

The use of telepathology in surgical pathology practice has received extensive attention and widespread acceptance. In high-income countries, digital revolution is toward sweeping the field and the practice of anatomic pathology; as laboratories are switching from the conventional microscope to digital slide scanners for primary diagnosis [32]. Studies from LMICs have highlighted good feasibility in implementing telepathology [21, 22]. In Tanzania, there are indeed several research projects on telepathology and these have given us important insights on feasibility and clinical utility [13–15]. However, poor participation of indigenous researchers suggests an important limitation in these studies. Moreover, sustainability factors have not been critically evaluated. For instance, to date, there have been no large studies showing the success of telepathology which have involved various health centers within the country. For successful performance of this promising technological innovation, several factors should be considered. These include operating costs, data security as well as user acceptance. Additionally, continuous training of pathologists regarding principles and limitation of digital pathology, sustainable internet connectivity, continuous bi-directional communication, and support from the institutional leadership are key to the success of telepathology programs [33–36]. A study comparing the cost for telepathology and a visiting pathologist services has revealed that establishing and running telepathology services were cheaper compared to hiring a visiting pathologist [37]. The main costs of the telepathology service were related to installation of the technology, whereas the main costs of the visiting pathologist service were payroll costs. A neuropathology consultation study involving Muhimimbili National Hospital (Tanzania) and University of Colorado (USA) documented the initial high cost of telepathology equipment, especially robotic and WSI telepathology as a challenge [38].

The results of this study indicate that telepathology can be used not only for primary diagnosis, but also as an international consultation platform that can improve patient care in Tanzania by facilitating access to pathology expertise. Once introduced, telepathology can revolutionize pathology services, as it is an important emerging adjunct to conventional light microscopy. It will enable remote diagnosis and improve collaboration among pathologists.

The important strength of the present study is that it is one among few validation studies carried out in Tanzania. However, the study has some limitations. Firstly, the time needed to establish diagnoses on both conventional and telepathology platform was not recorded. Therefore, the actual turn-around times could not be established. Secondly, cost analysis of implementing the telepathology program was out of the scope of this study. Thirdly, infrastructural obstacles in using immunohistochemistry in limited resources setting; the intra and interobserver variablility most likely would have been improved by the use of p16 immunohistochemistry. Fourth, our study included exclusively cervical biopsies with a relatively small sample size. Thus, for generalizability purposes, larger studies involving many pathologists and with a wide range of organ systems (to assess the clinical impact and the effect of telepathology on surgical pathology) are recommended. Lastly, but not least, despite good agreement rates between telepathology and conventional microscopy established in this study, the findings should be interpreted cautiously since the kappa statistics had rather wide confidence intervals. In addition, there are some disputes of the value of using kappa statistics due to difficulties in interpreting indices of agreement [39–42]. Therefore, setting an acceptable value of kappa should depend on the clinical context.

This study focused on the performance of telepathology in relation to cervical cancer diagnosis. The fight against cervical cancer has been given increasing priority lately with the WHO Director-General announcing a global call for action towards the elimination of invasive cervical cancer as a public health problem. Through cost-effective, evidence based interventions, including human papillomavirus vaccination of girls, screening and treatment of precancerous lesions, and improving access to diagnosis and treatment of invasive cancers [43]. In that relation it has been acknowledged that there is a need for more innovative technologies for detection of CIN2+ [44]. Our results illustrate how telepathology can be used to increase access to appropriate diagnostic service and identification of precancerous lesions and cervical cancer. This may lead to improved diagnosis and treatment and ultimately improved survival of women who are at risk of dying from cervical cancer.

## Conclusions

In summary, our study demonstrate that diagnoses of cervical premalignant and malignant lesions in biopsies are highly reproducible using both glass and digital formats of the slides, implying that telepathology is non-inferior to conventional light microscopy for primary diagnosis. The comparable diagnostic concordance between Tanzanian and Danish pathologists suggests that telepathology service can be a reliable tool to support primary cancer diagnosis in resource-limited settings that have limited numbers of pathologists. Studies assessing the utility of telepathology on cytological specimens including Pap smears are recommended. Once validated, the technology has the potential to scale up cancer control in Tanzania.

## Supporting information

S1 Data Deidentified raw dataset for the study participants (scml.dta). (DTA)

S1 Appendix Definition of the key terminologies [23]. (DOCX)
