# Supplementary material for: The role of telepathology in diagnosis of pre-malignant and malignant cervical lesions: Implementation at a tertiary hospital in Northern Tanzania
Source: PLoS One. 2022 Apr 14;17(4):e0266649. doi: 10.1371/journal.pone.0266649 (PMC9009664; doi:10.1371/journal.pone.0266649)
Supplement: S1 Appendix — (DOCX) [file pone.0266649.s002.docx]

**Appendix 1: Definition of the key terminologies [24]**

| Concordance | Essentially complete agreement between the two diagnoses |
| --- | --- |
| Partial concordance | A difference in diagnosis that would not be associated with a difference in patient care |
| Discordance | A difference in diagnosis that would be associated with a difference in patient care |
| Conventional diagnosis | The diagnosis (based on review of glass slides on light microscope) used for patient care |
| Telepathology diagnosis | The diagnosis based on review of whole slide images on telepathology platform. |
| Wash-out period | The interval between conventional (initial) and review (telepathology) diagnoses |
